# Identification and characterization of two *SERPINC1* mutations causing congenital antithrombin deficiency

**DOI:** 10.1186/s12959-022-00443-6

**Published:** 2023-01-09

**Authors:** Han-lu Wang, Dan-dan Ruan, Min Wu, Yuan-yuan Ji, Xing-xing Hu, Qiu-yan Wu, Yan-ping Zhang, Bin Lin, Ya-nan Hu, Hang Wang, Yi Tang, Zhu-ting Fang, Jie-wei Luo, Li-sheng Liao, Mei-zhu Gao

**Affiliations:** 1 Fujian Provincial Hospital, Shengli Clinical Medical College of Fujian Medical University, Fuzhou, 350001 China; 2 Department of Cardiovascular Medicine, Fujian Provincial Hospital, Fuzhou, 350001 China; 3 Department of Cardiovascular Surgery, Fujian Provincial Hospital, Fuzhou, 350001 China; 4 Department of Interventional Radiology, Fujian Provincial Hospital, Fuzhou, 350001 China; 5 Department of Traditional Chinese Medicine, Fujian Provincial Hospital, Fuzhou, 350001 China; 6 Department of Hematology, Fujian Provincial Hospital, Fuzhou, 350001 China; 7 Department of Nephrology, Fujian Provincial Hospital, Fuzhou, 350001 China

**Keywords:** Antithrombin, Deficiency, *SERPINC1*, Mutation, Vein thrombosis

## Abstract

**Background:**

Antithrombin (AT) is the main physiological anticoagulant involved in hemostasis. Hereditary AT deficiency is a rare autosomal dominant thrombotic disease mainly caused by mutations in *SERPINC1*, which was usually manifested as venous thrombosis and pulmonary embolism. In this study, we analyzed the clinical characteristics and screened for mutant genes in two pedigrees with hereditary AT deficiency, and the functional effects of the pathogenic mutations were evaluated.

**Methods:**

Candidate gene variants were analyzed by next-generation sequencing to screen pathogenic mutations in probands, followed by segregation analysis in families by Sanger sequencing. Mutant and wild-type plasmids were constructed and transfected into HEK293T cells to observe protein expression and cellular localization of *SERPINC1*. The structure and function of the mutations were analyzed by bioinformatic analyses.

**Results:**

The proband of pedigree A with AT deficiency carried a heterozygous frameshift mutation c.1377delC (p.Asn460Thrfs*20) in *SERPINC1* (NM000488.3), a 1377C base deletion in exon 7 resulting in a backward shift of the open reading frame, with termination after translation of 20 residues, and a different residue sequence translated after the frameshift. Bioinformatics analysis suggests that the missing amino acid sequence caused by the frameshift mutation might disrupt the disulfide bond between Cys279 and Cys462 and affect the structural function of the protein. This newly discovered variant is not currently included in the ClinVar and HGMD databases. p.Arg229* resulted in a premature stop codon in exon 4, and bioinformatics analysis suggests that the truncated protein structure lost its domain of interaction with factor IX (Ala414 site) after the deletion of nonsense mutations. However, considering the AT truncation protein resulting from the p.Arg229* variant loss a great proportion of the molecule, we speculate the variant may affect two functional domains HBS and RCL and lack of the corresponding function. The thrombophilia and decreased-AT-activity phenotypes of the two pedigrees were separated from their genetic variants. After lentiviral plasmid transfection into HEK293T cells, the expression level of AT protein decreased in the constructed c.1377delC mutant cells compared to that in the wild-type, which was not only reduced in c.685C > T mutant cells but also showed a significant band at 35 kDa, suggesting a truncated protein. Immunofluorescence localization showed no significant differences in protein localization before and after the mutation.

**Conclusions:**

The p.Asn460Thrfs*20 and p.Arg229* variants of *SERPINC1* were responsible for the two hereditary AT deficiency pedigrees, which led to AT deficiency by different mechanisms. The p.Asn460Thrfs*20 variant is reported for the first time.

**Supplementary Information:**

The online version contains supplementary material available at 10.1186/s12959-022-00443-6.

## Background

The human anticoagulant system works with the fibrinolytic system to maintain the balance between bleeding and coagulation. Antithrombin (AT), a 432-amino-acid single-chain glycoprotein synthesized and secreted in the liver, is the most important physiological anticoagulant in plasma [1]. AT is a member of the anti-serine protease inhibitor superfamily and binds to serine residues in the active centers of thrombin and coagulation factors IXa, Xa, XIa, and XIIa (FIXa, FXa, FXIa, and FXIIa), thereby "sealing" and inactivating their active centers with a pronounced anticoagulant effect [2]. In addition, AT inhibits activated factor VIIa (FVIIa) by accelerating the dissociation of the FVIIa/tissue factor (FVIIa/TF) complex and preventing its recombination, thereby preventing coagulation initiation [3]. Physiologically, the anticoagulant activity of AT is low, but when combined with high-affinity heparin, it can be rapidly amplified 1000-fold [4]. Owing to the unique anticoagulant mechanism and extensive anticoagulant activity of AT, a mild deficiency increases the risk of thrombosis, whereas a deficiency increases the risk of venous thrombosis 5–50-fold [5]. This is a major risk factor for thrombophilia, conferring a much higher risk than protein C or protein S deficiency.

Currently, there is no consensus on the minimum AT level defining a deficiency, but AT levels of at least 70% are necessary to effectively inhibit the coagulation cascade [6]. AT deficiency can be categorized as hereditary or secondary. Hereditary AT deficiency is a rare autosomal incompletely dominant disease characterized by clinical manifestations of recurrent thrombosis, of which lower extremity deep venous thrombosis and pulmonary embolism are the most common. There is a population incidence of approximately 1/5000 to 1/500 [7, 8], but the incidence may be about 1/200 to 1/20 in patients with venous thrombosis [9]. This genetic defect is mainly caused by mutations in *SERPINC1*, which encodes AT. *SERPINC1* is located on chromosome 1 q23.1–25, is 13.5 kb in length, and consists of seven exons and six introns [10]. Furthermore, *SERPINC1* is highly susceptible to alterations, and even small changes in its nucleotide sequence can cause severe structural and functional changes promoting thrombosis [11]. Most clinical cases are heterozygous because it is difficult for homozygotes to survive, and most die during embryonic development. Homozygous deletion of *SERPINC1* in mouse models has been demonstrated to result in severe thrombosis and hemorrhage, culminating in embryonic death [12, 13].

The *SERPINC1* mutation profile is highly heterogeneous, with 409 *SERPINC1* pathogenic mutations recorded in the HGMD database as of April 2021 (http://www.hgmd.cf.ac.uk/ac/gene.php gene = *SERPINC1*). Most of these mutations alter a single protein structural unit in AT, thereby disrupting its ability to inhibit coagulation [14]. Martinez-Martinez et al. found that genetic mutations may affect the mobile domains of conformational instability induced by AT, leading to protein polymerization associated with severe clinical phenotypes [15]. The variant types include point mutations, splicing, and small fragment insertion/deletion mutations [16, 17]. More than 90% of these mutations are point mutations [18]. The HGMD database showed that missense/nonsense mutations accounted for 55.0% of all mutations, frame-transfer mutations caused by small fragment deletions or insertions accounted for 26.5%, splicing mutations accounted for 7.6%, and other types of mutations accounted for 10.9%. In this study, two pedigrees with AT deficiency and recurrent venous thrombotic episodes were screened for causative genes to analyze the relationship between clinical phenotypes, gene variants, and cell function.

## Methods

### Study subjects

**Fig. 1:**
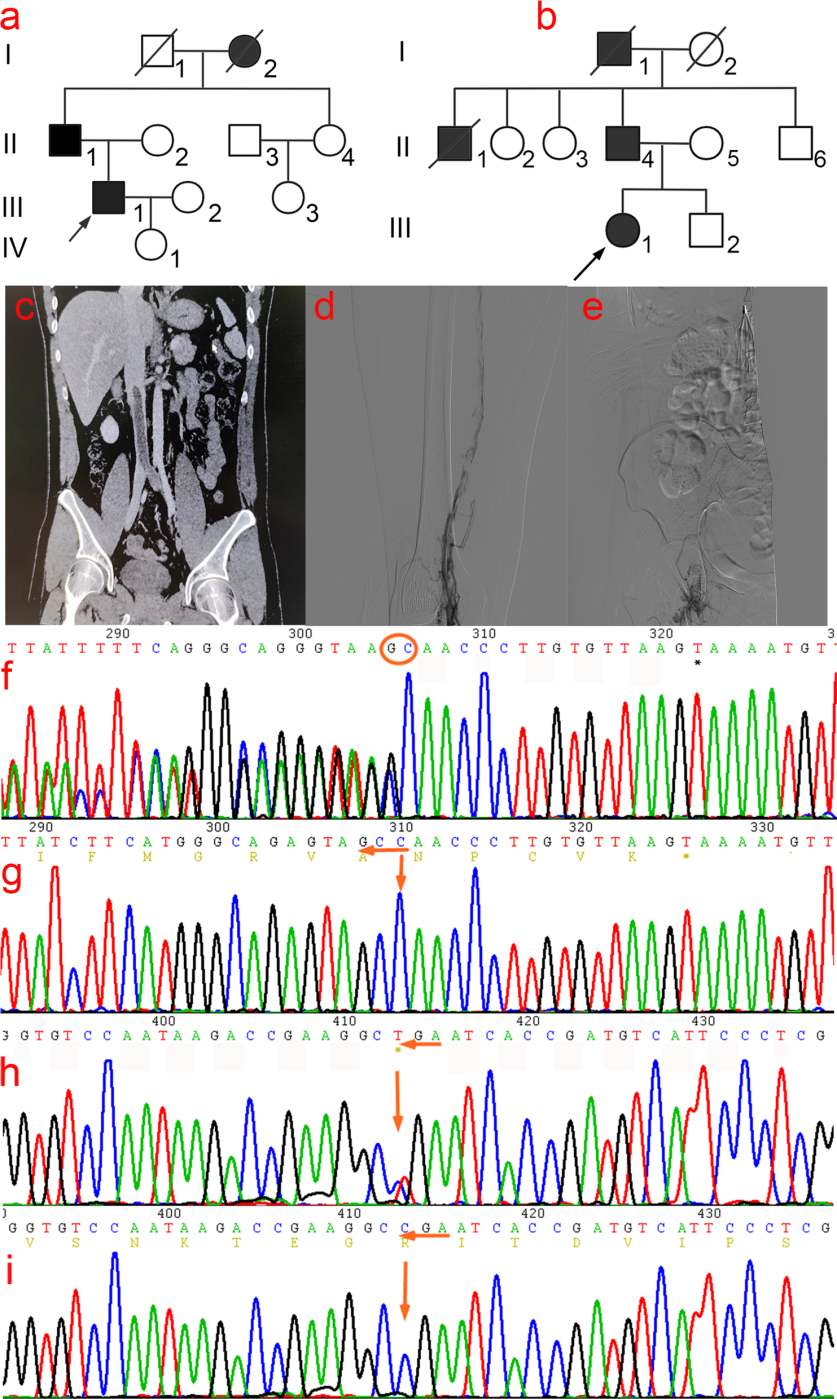
(**a**–**b**) Two pedigree profiles of hereditary antithrombin deficiency. (**c**) Computed tomographic angiography (CTA) findings of the small bowel mesenteric vessels in proband (III1) of Pedigree A showed thrombosis in the inferior vena cava, left common iliac vein, external iliac vein, and left external iliac vein. (**d**–**e**) Digital subtraction angiography findings of the proband (III1) in Pedigree B showed thrombosis of the right superficial femoral vein-common femoral vein and distal right external iliac vein. (**f**) A heterozygous mutation *SERPINC1* c.1377delC: p.Asn460Thrfs*20 was found in the proband of Pedigree A (III1). (**g**) Wild-type at c.1377C. (**h**) A heterozygous mutation *SERPINC1* c.685C > T: p. (Arg229*) was found in the proband of Pedigree B. (**i**) Wild-type of c.685C

Pedigree A (Fig. 1a): the proband (III1), a 31-year-old Han Chinese male, presented to the hospital with complaints of left lower quadrant pain that had lasted for six days. The patient was previously diagnosed with "deep venous thrombosis of the left lower limb" at another hospital 10 years ago and was administered anticoagulant and thrombolytic therapy. His parents were non-consanguineous, his father (II1) had a history of lower extremity deep venous thrombosis, and his grandmother (I2) died of pulmonary embolism.

Pedigree B (Fig. 1b): The proband (III1), a 36-year-old, Han Chinese female, had an intrauterine pregnancy at 16 + 5 weeks of gestation in G2P0. She presented to the hospital with complaints of right lower extremity pain lasting > 20 days and aggravation for 4 days. She had previously undergone thyroid nodule resection at another hospital 12 years previously. Her parents were non-consanguineous; her father (II4) had a history of lower extremity deep venous thrombosis, and her uncle (II1) and grandfather (I1) died of pulmonary embolism.

### Clinical phenotyping

Clinical examination data of probands and related family members, such as routine blood tests, coagulation function, liver and kidney function, and other laboratory tests related to thrombophilia, were collected. Protein S activity (PS: A) was measured using coagulation. Protein C (PC: A) and antithrombin (AT: A) activities were measured using the chromogenic substrate method.

### Extraction of genomic DNA

Genomic DNA from the peripheral blood of the proband and their family members was extracted using the QIAGEN DNA Blood Mini Kit (QIAGEN Co., Ltd.) following the manufacturer’s instructions. Ethical approval was granted by the Ethics Committee of Fujian Provincial Hospital, and all the investigated family members or their guardians signed informed consent forms.

### Mutant mapping and screening strategy

TargetSeq® liquid probe hybridization capture technology, independently developed by iGeneTech® (Beijing, China) was used to establish a genomic DNA library. Target gene exons and DNA adjacent to the cleavage region were captured and enriched, and the enriched DNA fragments were sequenced using the Illumina X10 platform. The target genes were *PROS1*, *PROC*, *SERPINC1*, *HABP2*, and *MYHFR*. The quality control indicators of the sequencing data were as follows: average effective sequencing depth of the target region ≥ 100 × , and > 95% of sites with an average sequencing depth of the target region > 20 × . Sequencing data were aligned to the human reference sequence UCSC hg19 using the Burrows–Wheeler Alignment, and mutations were screened and annotated using several databases such as 1000Genomes, gnomAD, ESP6500, HGMD, and ClinVar. SIFT (http://sift.jcvi.org/), PolyPhen-2(http://genetics.bwh.harvard.edu/ppH2/), Mutation Taster (http://mutationtaster.org/), and other software packages were used to predict the pathogenicity and toxicity of mutations. Protein structural analysis was performed using the Swiss PDB viewer and the artificial intelligence (AI) software AlphaFold2. Primers were synthesized by Synbio Technologies (Suzhou, China). Premier 5.0 was used to design primers for the upstream and downstream positions of the sequence where the target mutation site was located, and the target region was amplified. The corresponding suspected disease-causing mutations were verified by Sanger sequencing on an ABI 3500 Dx platform. The amplified fragment length of c.1377delC: p.Asn460Thrfs*20 at the mutation point *SERPINC1* (NM000488.3) was 505 bp and the primers used were F: ACAACTTGAAGGCATTTTACC; R: GTAGAAGTAGGATTCTGGAGGGAAT. The amplified fragment length of the sequence in which T: p.Arg229* resides was 510 bp, and the primers used were: F: CTCCAGGGCCATTCTGAGTA; R: CCTGGCACATGCCTTGGAAATT; annealing temperature, 61 °C.

### Construction and identification of SERPINC1 wild-type and p.Asn460Thrfs*20 and p.Arg229* mutants plasmids

**Fig. 2:**
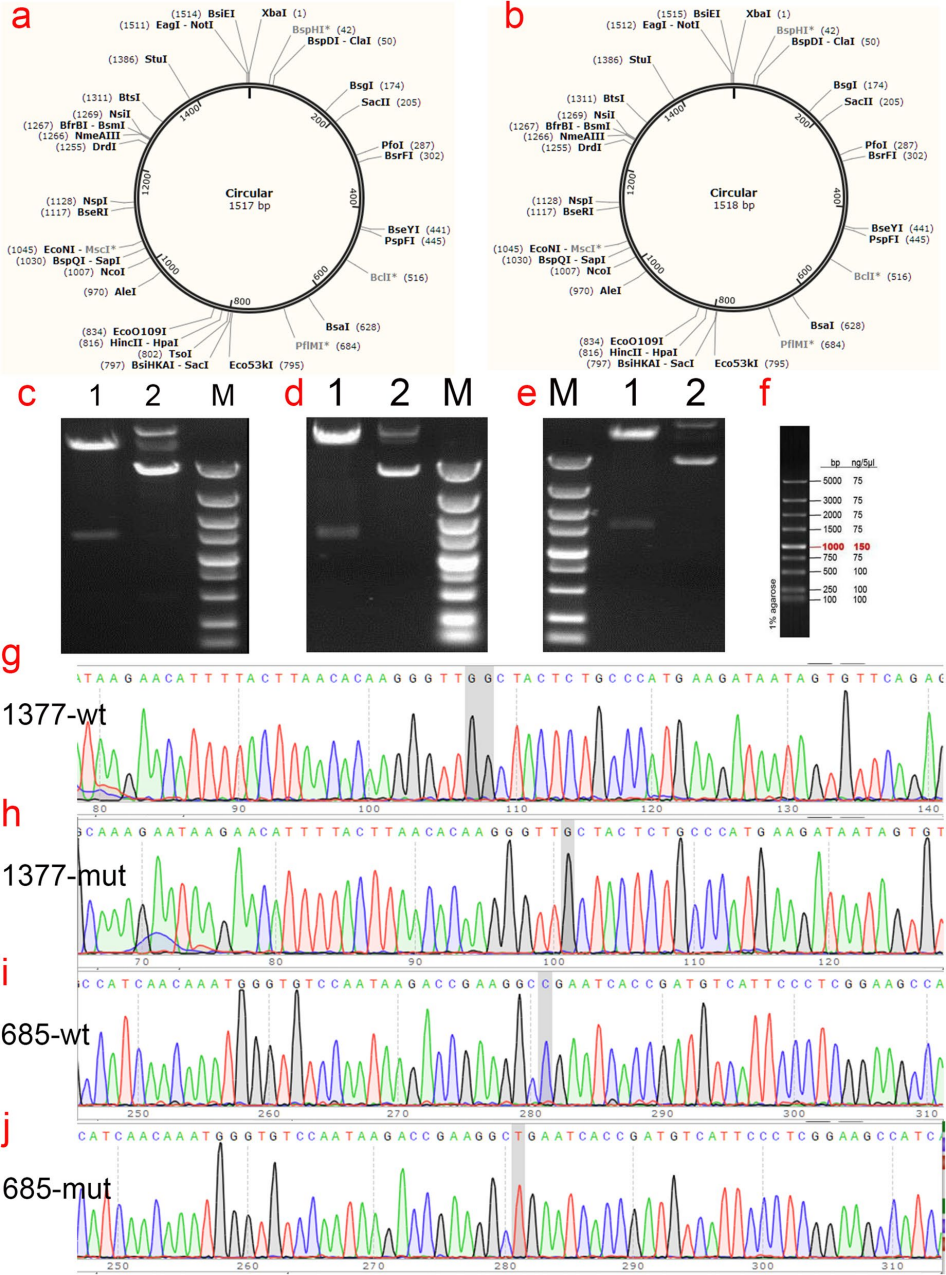
Gene cloning and plasmid vector construction. (**a**) Schematic diagram of mutant c.1377delC plasmid vector: pCDH-CMV-*SERPINC1* (c.1377delC) -EF1-copGFP-T2A-Puro. (**b**) Schematic diagram of mutant c.685 T plasmid vector: pCDH-CMV-*SERPINC1* (c.685C > T) -EF1-copGFP-T2A-Puro. (**c**) Electropherogram of wild-type plasmid digested with NotI/XhoI; (**d**) Electropherogram of mutant c.1377delC plasmid digested with NotI/XhoI; (**e**) Electropherogram of mutant c.685C > T plasmid digested with NotI/XhoI, Lane 1: undigested electropherogram, Lane 2: electropherogram after double digestion, Lane 3: DNA marker. (**g**-**j**) Sequencing of the clone plasmid of the *SERPINC1* gene

The plasmid synthesis scheme is shown in Fig. 2. The expression vector used was pCDH-CMV-MCS-EF1-copGFP-T2A-puro to synthesize the *SERPINC1* gene with double restriction sites: NotI/XhoI (Fig. 2c- 2f). Next, various mutant plasmids were constructed as follows: wild-type plasmid 1, pCDH-CMV-*SERPINC1* (WT) -EF1-copGFP-T2A-Puro with double restriction sites NotI/XhoI; mutant plasmid 2, pCDH-CMV-*SERPINC1* (c.1377delC)-EF1-copGFP-T2A-Puro, double restriction sites NotI/XhoI, including the 1377delC mutation site of *SERPINC1*; mutant plasmid 3, pCDH-CMV-*SERPINC1* (c.685C > T)-EF1-copGFP-T2A-Puro, restriction site NotI/XhoI, encompassing the 685C > T mutation site of *SERPINC1*. Amplification, validation, and sequencing of the target genes were performed. *SERPINC1* (WT), *SERPINC1* (1377delC), and *SERPINC1* (685C > T) gene cloning and PCR primer synthesis were performed by Wuhan GeneCreate Biological Engineering.

### Cell transfection

HEK293T cells were collected by trypsin digestion and plated on 10 cm cell culture dishes at a density of 1–2 × 10^7^ cells/plate with DMEM complete medium. The total area occupied after cell attachment was 80–90% of the dish area. Transient transfection was initiated after complete cell attachment by incubation in a 37 °C incubator containing 5% CO_2_ for 8–24 h according to cell attachment. TurboFect-DNA Mix was prepared according to TurboFect (R0531, Thermo, GER) instructions. Briefly, 1 µg/plasmid and 2 µL TurboFect were added to 200 µL Opti-Medium, gently mixed, and incubated at room temperature for 15 min. Next, TurboFect-DNA Mix was added to the culture dish and the complete medium was replaced after 12 h. The culture was incubated for 48 h. The cells were observed under a microscope, and the medium was collected for the next step.

### Quantitative real-time PCR

Total RNA was isolated from HEK293T cells using the TRIPURE ISOLATION REAGENT kit (11,667,165,001; Roche, Switzerland). Reverse transcription and qRT-PCR were used to detect differences in the transcript levels of *SERPINC1*. First-strand cDNA was synthesized from the total RNA using a HiFiScript RT reagent kit (CW2020M, CWBIO, Beijing, CHN). mM dNTP Mix 2.5 mM 4 µL; Primer Mix 2 µL (Primers are listed in Table 1); RNA Template 7 µL; 5 × RT Buffer 4 µL; 1 × DTT 0.1 M 2 µL; and 200 U/µL 10 mM HiFiScript; RNase-Free Water was added to make a total volume of 20 µL. The solution was vortexed, briefly centrifuged, and incubated at 42 °C for 50 min and 85 °C for 5 min. Reverse-transcribed cDNA was diluted 20-fold for RT-qPCR, which was performed using a Roche LightCycler 480 for 40 cycles.

**Table 1:** Primers for quantitative real-time PCR

primer name	primer sequence
hSERPINC1 qPCR F	5`-AGGCTGATGGAGAGTCGTGT-3`
hSERPINC1 qPCR R	5`-CCAGCATCATCTCCTCCAAT-3`
hGAPDH F	5`-CAAGGTCATCCATGACAACTTTG-3`
hGAPDH R	5`-GTCCACCACCCTGTTGCTGTAG-3`

### Western blotting assay

Total protein from HEK293T cells was cultured, lysed (RIPA lysis buffer), extracted, and AT protein expression was detected. Protein samples and maker were loaded into electrophoresis gel wells, wet-transferred to polyvinylidene fluoride (PVDF) membranes after electrophoresis, and soaked in 5% skimmed milk prepared in Tween 20 (TBST) at room temperature for 1 h. The membranes were washed once and incubated with primary antibody solution (Flag antibody (F1804, SIGMA, USA), 1:3000; anti-a-Tubulin antibody (ab7291, Abcam, UK), 1:3000) diluted in 5% bovine serum albumin (BSA) overnight at 4 °C. After washing the membranes three times, 5% BSA was added to dilute the horseradish peroxidase-labeled secondary antibody (goat anti-mouse IgG; ab6789, Abcam, UK), and the samples were incubated in a shaker at room temperature for 1 h. The sections were visualized after five washes with TBST and one wash with ddH2O.

### Enzyme linked immunosorbent assay (ELISA) of AT in HEK293T cell cell supernatants

AT levels secreted to the conditioned medium of HEK293T cells were determined by a commercial ELISA following the indications of the manufacturer (ab222507, Abcam, UK). The parameters of the microplate reader (Varioskan Lux, Thermo Fisher Scientific, Waltham, MA, USA) were set. A microplate reader was used to measure the optical density of the substrate solution at 450 nm immediately after termination of the reaction. A standard curve was drawn, and AT content were calculated in the samples to be measured.

### Immunofluorescence localization assay

Transfected HEK293T cells were fixed, permeabilized, blocked, and incubated overnight at 4 °C with AT primary antibody (diluted 1:200). Cells were washed three times with PBS before the addition of a fluorescent secondary antibody (1:500 dilution) and incubated at room temperature for 2 h in the dark. Next, the cells were washed three times with PBS and incubated with DAPI at room temperature for 5 min. After three 3-min washes with 1 × PBS, images were obtained using a confocal laser-scanning microscope (Nikon A1, JPN).

### Statistics

GraphPad Prism (v. 6.02) statistical software was used to analyze the experimental data. The F test was used for variance among three groups, the least significant difference test was used for two-to-two comparison, and the unpaired *t*-test was used for two group comparisons. Data are presented as mean ± SEM, *P* < 0.05 was considered a significant difference.

## Results

### Clinical phenotype

Pedigree A (Fig. 1a): Male proband III1 presented with left lower extremity deep venous thrombosis at age 21 and left lower quadrant pain at age 31. After admission, computed tomography angiography of the mesenteric vessels of the small bowel was performed. This revealed low-density filling defects in the inferior vena cava and left common and external iliac veins in the venous phase, nodular dense shadows in the left external iliac vein, multiple tortuous and thickened subcutaneous veins in the lower abdomen, and communication with the bilateral external iliac veins, which tended to form emboli (Fig. 1c). Color Doppler ultrasound of the deep vein of the left lower limb revealed thrombosis of the left common iliac vein and an old thrombus extending from the left common femoral vein to the superficial femoral vein, with extensive recanalization. Laboratory tests showed AT, 35.0% (normal range, 75–125%); PC, 81.2% (normal range, 70–140%); and PS, 45.6% (normal range, male 75–130%, female 52–118%) (Table 2). Furthermore, anticardiolipin, antiphospholipid, and antinuclear antibodies showed no abnormalities. Patient II1 had a history of lower extremity deep venous thrombosis, and his ratio Ac/Ag also decreased significantly (30.4%), whereas patient I2 died of pulmonary embolism.

**Table 2:** Coagulation function indexs of proband and family members in hereditary antithrombin deficiency family

Family members	PT (s)	APTT (s)	TT (s)	Fg (g/L)	D-dimer	PS(%)	PC(%)	AT-III(%)
**Proband A(III1)**	11.70	25.10	14.80	4.17	6.66	23.20	78.20	36.20
**II1**	10.90	23.80	13.90	2.82	0.53	58.50	80.10	30.40
II2	11.20	24.60	16.50	2.10	0.23	63.20	123.30	90.50
II3	10.90	27.20	18.30	1.91	0.15	82.10	98.80	103.60
II4	12.80	29.80	15.10	2.32	0.32	75.60	103.00	99.20
III2	10.40	24.50	19.30	1.85	0.12	82.40	114.00	108.10
III3	9.80	26.10	18.70	2.10	0.25	83.80	89.60	111.90
IV1	13.00	26.50	19.20	2.70	0.30	89.20	128.30	103.70
**Proband B(III1)**	11.20	24.90	13.60	6.45	11.90	36.10	85.10	38.60
**II4**	12.10	25.90	14.50	2.30	0.48	61.20	79.00	49.80
II2	9.80	23.80	15.90	2.35	0.35	73.60	100.50	90.60
II3	11.50	26.50	16.10	1.92	0.27	78.10	115.30	87.90
II5	12.30	23.10	17.20	2.56	0.33	88.50	117.10	103.50
II6	11.60	28.70	16.60	1.99	0.23	65.70	97.20	99.70
III2	11.80	24.60	15.20	2.11	0.21	83.30	109.80	107.80
reference value	9.9–12.9	23.3–32.5	14–21	1.8–3.5	0–0.55	Male:75–130 Female:52–118	70–140	75–125

*Note*: *PT* prothrombin time, *APTT* activated partial thromboplastin time, *TT* thrombin time, *Fg* fibrinogen, *PS* protein S, *PC* protein C, *AT* antithrombin III

Pedigree B (Fig. 1b): Proband III1, a 36-year-old Han Chinese woman, presented with right lower extremity pain in utero at 16 + 5 weeks of gestation, lasting for more than 20 days. After admission, color Doppler ultrasonography of the right lower extremity was performed. This revealed a hypoechoic filling from the right external iliac vein to the deep vein of the right lower extremity with no significant blood flow signals, suggesting thrombosis. Digital subtraction angiography showed difficulty ascending the contrast agent in the right superficial femoral vein, multiple filling defects in the lumen, considering thrombus filling, and no ascending contrast agent from the distal segment of the right external iliac vein, suggesting thrombosis (Fig. 1d- 1e). Laboratory tests showed AT, 37.5%; PC, 84.7%; and PS, 58.2% (Table 2); anticardiolipin antibody, antiphospholipid antibody, and antinuclear antibody showed no abnormalities. The proband still had recurrent thrombosis despite receiving anticoagulant therapy during hospitalization. Patient II4 had a history of deep vein thrombosis of the lower extremities, and patient II1 and I1 died of pulmonary embolism.

### Mutation screening and Sanger validation for Thrombophilia

In pedigree A, no large deletions or duplications were identified after deep data analysis of whole exome sequencing of the thrombophilic gene. A heterozygous frameshift mutation c.1377delC: p.Asn460Thrfs*20 (Fig. 1f- 1g) was found in the coding region of *SERPINC1* (NM000488.3) in the proband (III1). This mutation caused a loss of base 1377 in exon 7 of *SERPINC1*, leading to a frameshift of 21 amino acids. Successive amino acid sequence changes result in the premature termination of translation and affect the original function of the protein. Pathogenicity at this locus has not been reported in ClinVar or HGMD databases. As verified by Sanger sequencing, patients II1 and III1 carried the p.Asn460Thrfs*20 mutation pattern, whereas the other unaffected patients in the family did not carry this mutation in II2, II3, II4, III2, III3, and IV1. Co-segregation between the genotype and phenotype was observed in the family. Therefore, the frameshift mutation p.Asn460Thrfs*20 was considered a pathogenic mutation in this family and is reported here for the first time.

In pedigree B, no large deletions or duplications were identified after deep data analysis of whole exome sequencing of the thrombophilic gene. A heterozygous nonsense mutation c.685C > T: p.Arg229* (Fig. 1h- 1i) was identified in the coding region of *SERPINC1* (NM000488.3) in the proband (III1). The mutation caused premature termination of arginine (Arg) at the 229th amino acid residue, leading to the production of a truncated protein. Pathogenicity at this locus has been previously reported [19]. A mutation Taster score of 1 was predicted to be deleterious and affect protein function. As verified by Sanger sequencing, patients II4 and III1 carried the p.Arg229* mutation pattern, whereas unaffected patients in the family did not carry this mutation in II2, II3, II5, II6, and III2. Co-segregation between the genotype and phenotype was observed in the family.

### Cloning of SERPINC1 wild-type and p.Asn460Thrfs*20 and p.Arg229* gene mutants

The cloning and eukaryotic expression vectors of *SERPINC1*-WT, *SERPINC1* (Asn460Thrfs*20), and *SERPINC1* (Arg229*) were successfully constructed (Fig. 1). The fragments generated by NotI/XhoI digestion of *SERPINC1*-WT, mutant *SERPINC1* (Asn460Thrfs*20), and *SERPINC1* (Arg229*) were approximately 1500 bp, which was consistent with the design (Fig. 2). The constructed vector was verified by sequencing (Fig. 2g- 2j) and was transfected into HEK293T cells.

### Detection of transfection efficiency of pCDH lentiviral expression vector

**Fig. 3:**
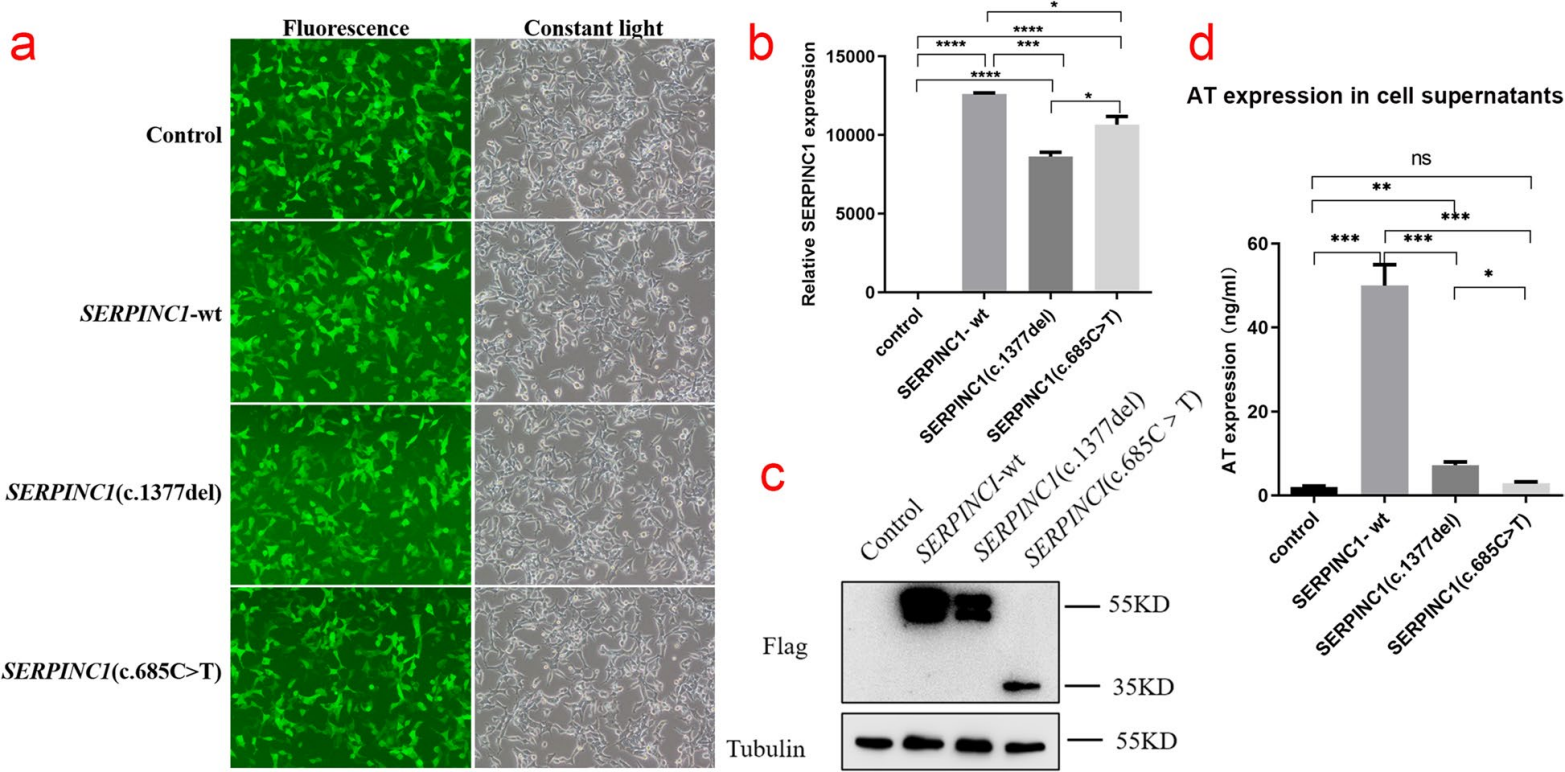
(**a**) Green fluorescent protein expression after transfection of cells carrying unloaded (Control), wild-type (*SERPINC1*-WT), Asn460Thrfs*20 mutant (*SERPINC1*-c.1377delC), and Arg229* mutant (*SERPINC1*-685C > T), suggesting that there was no significant difference in transfection efficiency among the groups. (**b**) Relative *SERPINC1* mRNA expression levels (*, *P* < 0.05, * *, *P* < 0.01, * *, *P* < 0.005, * *, *P* < 0.001) in HEK293T cells by Control, *SERPINC1*-WT, Asn460Thrfs20 mutant (*SERPINC1*-c.1377delC), and Arg229* mutant (*SERPINC1*-685C > T). (**c**) Western blotting was used to detect the expression of AT protein in HEK293T cells by Control, *SERPINC1*-WT, Asn460Thrfs*20 mutant (*SERPINC1*-c.1377delC) and Arg229* mutant (*SERPINC1*-685C > T), respectively. (**d**) Based on the enzyme-linked immunosorbent assay (ELISA) standard curve, the expression of Control, *SERPINC1*-WT, Asn460Thrfs*20 mutant (*SERPINC1*-c.1377delC), and Arg229* mutant (*SERPINC1*-685C > T) in the supernatant of HEK293T cells was calculated. (*, *P* < 0.05, * *, *P* < 0.01, * *, *P* < 0.005, * *, *P* < 0.001)

After 48–72 h of infection with HEK293T cells grown in the logarithmic phase with pCDH lentiviral empty load, pCDH-CMV-*SERPINC1* (WT) -EF1-copGFP-T2A-Puro, pCDH-CMV-*SERPINC1* (c.1377delC) -EF1-copGFP-T2A-Pur, and pCDH-CMV-*SERPINC1* (c.685C > T) -EF1-copGFP-T2A-Puro lentiviral expression vectors, the cells highly expressed green fluorescent protein (Fig. 3a), suggesting good growth status.

### Expression of SERPINC1-WT and mutants in HEK293T cells

Relative *SERPINC1* mRNA expression levels of the *SERPINC1-*WT, Asn460Thrfs*20 mutant and Arg229* mutant of *SERPINC1* gene in HEK293T cells were examined by qRT-PCR. Green fluorescent protein was used as an external reference to compare the relative differences in the *SERPINC1* mRNA expression group, and the results showed that *SERPINC1* mRNA expression was downregulated in Asn460Thrfs*20 and Arg229* mutants cells compared to that in wild-type cells (*P* < 0.05) (Fig. 3b). Western blotting was performed to detect the expression of the target protein, AT (Fig. 3c). The AT protein of wild-type cells showed a significant band at a molecular weight of approximately 55 kDa but not in the transfected empty vector group, indicating that this band was the target band. The AT protein in Asn460Thrfs*20 mutant cells also showed a distinct band at a molecular weight of approximately 55 kDa, but the expression of the AT protein was downregulated compared to that in the wild-type. AT protein expression was not only reduced in Arg229* mutant cells, but also showed a significant band at 35 kDa, suggesting a truncated protein. Meanwhile, the expression of AT in the cell culture medium supernatant was detected by ELISA (Fig. 3d). The results show that the expression level of AT protein in the cell culture medium supernatant and cell lysate in the Asn460Thrfs*20 and Arg229* variants groups was significantly lower than that in the wild-type group, which is consistent with the results of western blotting.

### Localization of wild-type AT and its mutants in cells

**Fig. 4:**
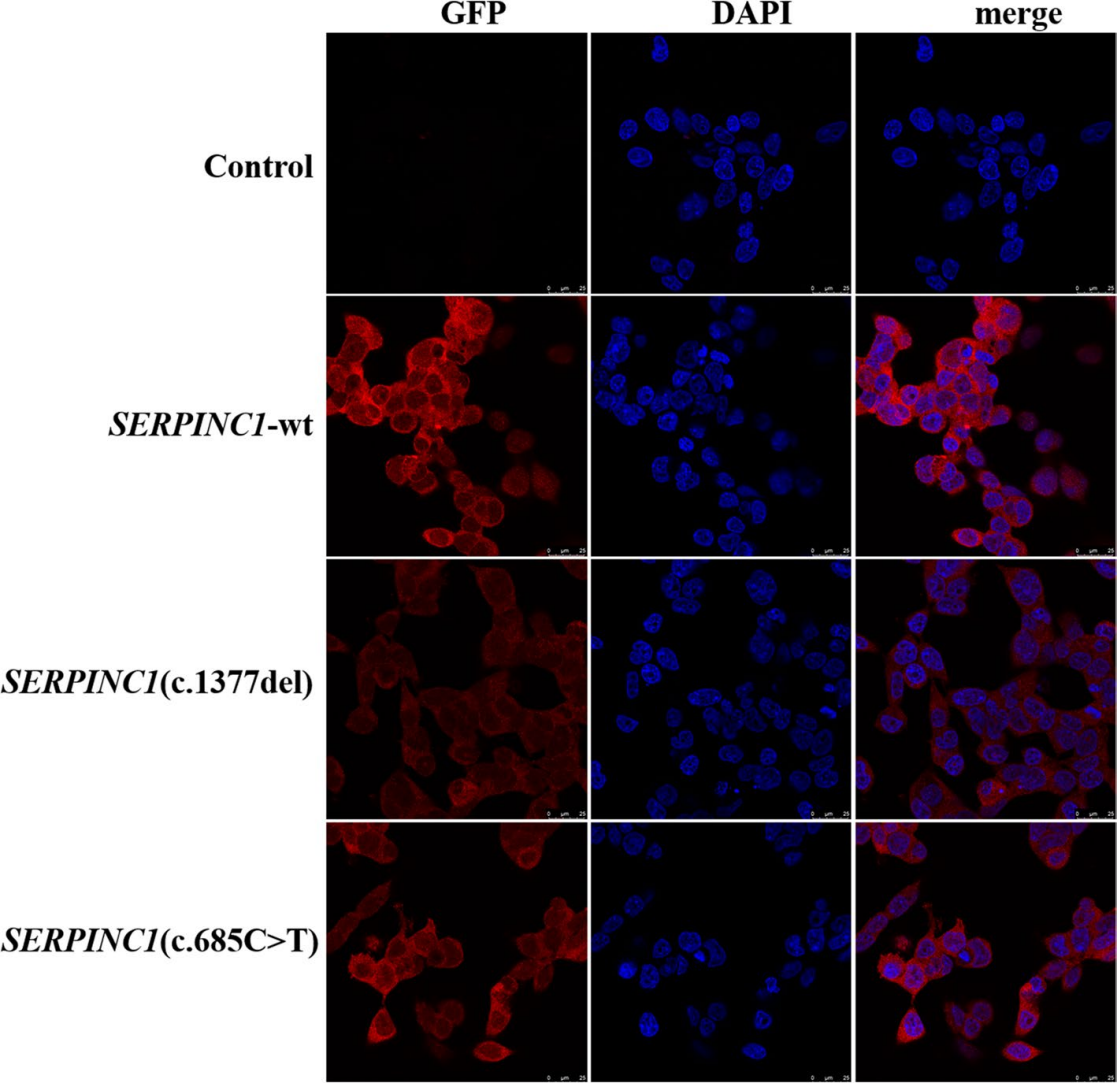
Immunofluorescence was used to detect the localization of AT protein in HEK293T cells carrying unloaded (Control), wild-type (*SERPINC1*-WT), Asn460Thrfs*20 mutant (*SERPINC1*-c.1377delC), and Arg229* mutant (*SERPINC1*-685C > T). Following *SERPINC1* (c.1377del) and *SERPINC1* (c.685C-T) mutation, AT protein expression level was attenuated, and the proteins were localized in the cytoplasm

Immunofluorescence localization experiments showed there was no significant difference in intracellular fluorescence localization before and after introduction of the *SERPINC1* Asn460Thrfs*20 and Arg229* mutants respectively, which all were distributed in the cytoplasm. However, the red fluorescence intensity of the Asn460Thrfs*20 and Arg229* mutants were significantly attenuated compared to that of the wild-type, suggesting that protein expression was similarly attenuated, consistent with the western blotting results (Fig. 4).

### Bioinformatics analysis

**Fig. 5:**
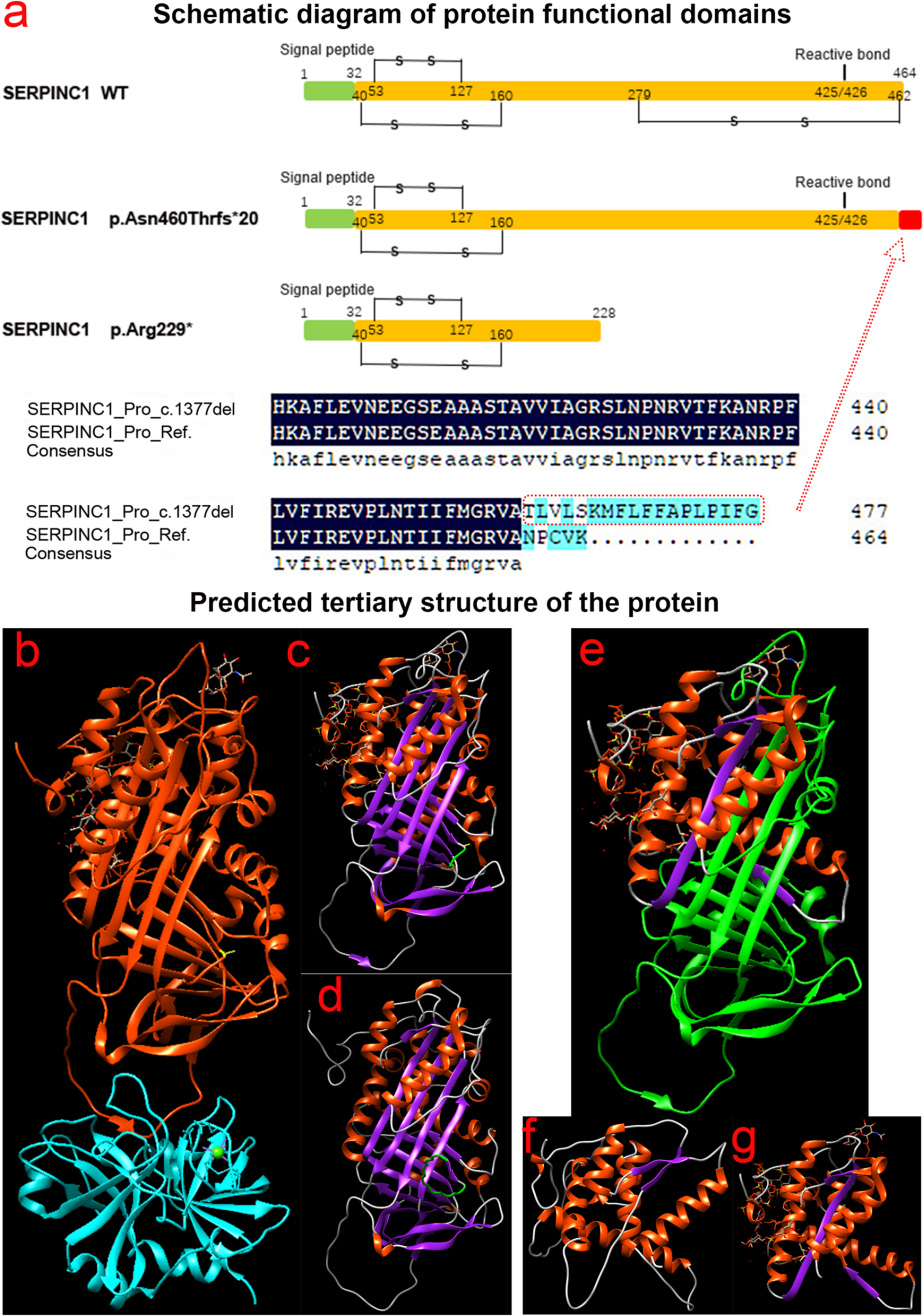
Prediction of structures of antithrombin (AT) protein mutants. (**a**) Schematic representation of functional domains of AT proteins and their variants. (**b**) Three-dimensional crystal structure diagram of AT protein versus coagulation factor IX (FIX) in Swiss-model database; red represents AT protein, green FIX protein, and yellow Asn460 (https://swissmodel.expasy.org/repository/uniprot/P00740?template=3kcg). (**c**) Swiss-model prediction of the three-dimensional structure of the Asn460Thrfs*20 mutant, with the yellow site represents Asn460 and the green sequence as the amino acid sequence missing from the frameshift mutation. (**d**) The three-dimensional structure of the Asn460Thrfs*20 mutant was predicted by Alphafold2 software, and the green sequence was the amino acid sequence that was generated after frameshift. (**e**) The sequence in green is the sequence deleted by the nonsense mutation Arg229*. (**f**) The deletion protein structure of the nonsense mutation Arg229* resulted in the loss of the domain interacting with FIX (Ala414 site). (**g**) Alphafold2 predicts the tertiary structure of a truncated protein with the nonsense mutation Arg229*

As shown in the schematic diagram of the AT protein and its variants (Fig. 5a), Swiss model software (https://swissmodel.expasy.org/repository/uniprot/P00740?template=3kcg) was used to observe the AT protein tertiary structure. In addition, Chimera observations (https://www.cgl.ucsf.edu/chimera/) were used to observe structural changes after the Asn460Thrfs*20 and Arg229* mutations (Fig. 3b, 3c, 3e, 3f). The mutation at position 460 caused breakage of the disulfide bond between Cys279 and Cys462 in the mutant protein, suggesting that this might affect the protein, whereas the domain interacting with FIX (Ala414 site) was lost at position 229. Alphafold2 predicted that the different residue sequences translated from Asn460Thrfs*20 after frameshift replaced the amino acid sequence deleted after frameshift mutation (Fig. 3d, 3g). In contrast, the Arg229* mutation results in a truncated protein.

## Discussion

Congenital AT deficiency was first reported by Egeberg in 1965 [20]. AT deficiency can be divided into two types based on AT activity and plasma antigen levels. Type I: Classical deficiency, characterized by impaired AT synthesis and a parallel decrease in plasma AT antigen and activity in patients. Its clinical manifestations are severe and usually associated with severe mutations in *SERPINC1*, which lead to a significant decrease in the level of AT in plasma owing to mutations that destabilize mRNA, misfolded proteins, and intracellular retention, or lead to abnormal degradation [21–23]. Type II mainly affects the domain function bound to thrombin or heparin, with normal plasma AT antigen content, but its activity is weakened [24], its clinical manifestations are mild, and its incidence is relatively high [25]. Moreover, the mutant forms are mostly missense mutations, which only affect the functional domain of AT [26–28]. Type II deficiency can be divided into three subtypes based on the location of the mutation: type II with reactive site defects (type IIRS), type II with heparin-binding defects (type IIHBS), and type II with pleiotropic defects (type IIPE). In type IIPE deficiency, mutations are commonly found in the highly conserved C-terminal hinge region (strands 1C–5 B) of AT [29–31]. Patients with type IIHBS have the lowest risk of VET [32]. Some patients with *SERPINC1* mutations may have normal AT activity. Carriers of the close hotspot mutations p.Val295Met, p.Arg294Leu, and p.Arg294Cys in *SERPINC1* have normal AT levels and anticoagulant activity in the Chinese population, as demonstrated in recombination models. However, mutation carriers have significantly increased endogenous thrombin potential. Patients with these variants have an approximately 20-fold increased risk of thrombosis [33].

Of all the patients with AT deficiency, 80% had defects in *SERPINC1*. Moreover, up to 20–30% of cases do not have any *SERPINC1* mutations, which may be associated with genetic variants, such as proteins encoding AT transcription, chaperones involved in AT folding, and post-translational modifications [34], up to 1/3 of these cases may be associated with N-glycosylation disorders of *SERPINC1* [35, 36]. N-glycosylation is an important post-translational modification associated with protein folding, function, and secretion [37]. *SERPINC1* encodes 32 amino acid signal peptides and 432 amino acid maturation proteins [38, 39]. The primary function of this signal peptide is to direct nascent polypeptide chains into the endoplasmic reticulum, followed by post-translational processing, including disulfide bond formation, N-glycosylation, signal peptide cleavage, and correct folding of the protein into a metastable conformation, which is required for the inhibitory activity of this serine protease inhibitor [22]. A portion of exons 2 and 3 encodes the heparin-binding site region [40]. A portion of exon 6 encodes the C-terminal region [41]. AT has a structure similar to serine protease inhibitors: three β-folds (A–C), nine α-helices (A–I), and a reactive center loop (RCL) [42]. It has two important functional structural regions: the protease inhibitory active region at the C-terminus and heparin-binding region at the N-terminus [43, 44]. Protease-binding sites are mainly distributed between A-β folds and C-β folds, and RCL can be formed by stretching outward on the molecular surface in this region. The spatial structure of RCL is complementary to that of the substrate-binding groove in the active site of serine proteases, thus exerting its role in protease inhibition. Heparin-binding sites are mainly distributed on the basic D helix, and they can bind pentameric polysaccharides in heparin polysaccharide chains and achieve tight binding with heparin by changing their conformation [45–48]. Lys 11, Arg 13, Arg46, Arg47, Lys 114, Lys125, and Arg129 have high affinity for heparin [49]. AT also has four glycosylation sites (Asn128, Asn167, Asn187, and Asn224) and three disulfide bonds (Cys40-Cys160, Cys53-Cys127, and Cys279-Cys462). Variations in these key residues can affect the structure and function of thrombin proteins [50]. During coagulation inhibition, RCL first identifies the substrate-binding site of the target protease, breaks its own Arg425-Ser426 peptide bond (P1-P1′), inserts the hinge of the broken RCL into the notch region of the A- β fold, and then covalently binds to Ser150 in the thrombin active center to form a stable AT-thrombin complex such that thrombin activity is lost or weakened. When the pentasaccharide sequence in the heparin molecule binds to AT, the hinge of the RCL is removed from the A-β fold, leaving the RCL completely exposed, which binds more thrombin and rapidly amplifies the anticoagulant activity of AT [51–54].

Modeling analysis of the AT protein suggested that this frameshift mutation caused breakage of the disulfide bond between Cys279-Cys462 and affected the structure and function of the protein. Mutations involving disulfide bond breakage have previously been reported to cause AT deficiency, including Cys279-Cys462 (Cys279Trp, Cys462Phe), Cys40-Cys160 (Cys160Tyr, Cys160Term, Cys160Trp), and Cys53-Cys127 (Cys53Ser, Cys53Term, Cys127Arg) [55]. In contrast, intramolecular disulfide bonds are essential for the correct folding, structural stabilization, and secretion of AT proteins [56]. For example, p.Cys127Arg causes the rupture of disulfide bonds between Cys53-Cys127, significantly slows the rate of AT secretion, and persists in the intracellular reticulum in the form of oligosaccharides, which cause AT deficiency [57]. Mutations 13,387-9delG and c.964A > T (p.Lys322*) cause breakage of the disulfide bond between Cys279 and Cys462, impairing secretion and structural stability of AT-truncated proteins degraded intracellularly [58, 59]. In addition, AT protein truncation caused by c.964A > T (p.Lys322*) led to the loss of the P1-P1′(Arg393-Ser394) bond, which does not act as an inactivated protease [58]. The Cys462Tyr mutation also breaks the disulfide bond between Cys247 and Cys430, triggering aberrant AT aggregation and leading to impaired secretion and eventual intracellular degradation [58]. Notably, p.Leu450fsX9 and p.Val458Cys462delGly variant, which are adjacent to Asn460 on exon7, may cause thrombopoiesis and AT activity of less than 50% [60, 61]. AT activity of less than 50% has been observed in many frameshift mutations in *SERPINC1*. When AT activity is less than 50%, the sensitivity of AT to heparin decreases rapidly, which increases observably the risk of thrombosis. Frameshift mutations often mean “loss of function”. Frameshift mutations can cause the lack of full-length proteins and damage multiple potential functional domains of AT, easily leading to severe decline in AT activity.

Bioinformatic analysis of the p.Arg229* variant suggested that this might affect protein viability because of the truncated product, while AT position 229 loses the domain interacting with FIX. Carriage of p.Arg229* has been found in patients with hereditary AT deficiency type I (Accession Number: CM920111) [19]. Approximately 12% of inherited disease-causing mutations in humans are nonsense mutations, which may lead to the production of truncated proteins that are hypoactive or inactive as well as gain-of-function or dominant-negative effects [62]. The p.Glu271* mutation is associated with recurrent DVT, cerebral artery thrombosis, and pulmonary embolism, and the p.Arg391* mutation is associated with cerebral venous sinus thrombosis [58, 63]. Modeling analysis of the AT protein revealed that the mutated AT protein lost its domain when interacting with FIXa. Heparin-binding AT action involves two distinct mechanisms: allosteric activation and bridging [64, 65]. Among these, FIXa and FXa are the main target proteases that are rapidly inhibited by heparin through the allosteric activation of AT [66, 67]. Heparin promotes the interaction of exosites in the RCL of AT with FXa and FIXa by mediating allosteric activation of AT. Furthermore, the external binding site is a key factor in determining the response of AT to FXa and FIXa [48, 68]. Several external binding sites for FIXa and FXa are found in the region consisting of amino acids 230–310 of AT, such as Asn233, Arg235, Glu237, Tyr253, and Glu255 [69]. It has also been suggested that the extracellular binding site of AT is on the 232–255 contiguous loop, which forms a folding chain of 3 and 4C-β that can bind 143–153 and Glu74 on the autolytic loop residues of FIXa [38]. Therefore, Arg229, which is located near this region, may also belong to this category, which led us to consider the impact of such positional mutations. Tyr253 and His319 are critical sites for the external binding of AT. They specifically bind to Arg150 on FXa and FIXa, and mutations in these two locations could reduce the response of AT to FXa and FIXa by 10–200-fold [70, 71]. Adjacent exosites Tyr285 and Glu287 enhance the interaction of heparin-allosterically activated AT with target proteins [33]. This implies that Arg229 may be associated with an external binding site on the RCL of AT, and mutations at this position may reduce the ability of AT to react with FIXa and enhance the activity of FIXa. FIXa is a key factor in determining the intensity and efficiency of coagulation reactions [72, 73]. The F helix in the AT protein structure belongs to a highly conserved region and is related to the stability of AT protein conformation [74]. The AT Rouen-VI (Asn187Asp) variant of the F helix affects the stability of the A–β fold and forms polymers that are retained intracellularly [75]. The Gly228 residue is located at the end of the F helix, and when replaced, the AT protein conformation is unstable, resulting in polymer formation, and thus, is not degraded and trapped in the cell [76]. Similarly, Arg229 is also on the F helix, and mutations at this position mostly affect the stability of the AT conformation, occasionally forming polymers that may be associated with slowing the rate of RCL insertion into the A-β-fold or disruption of the stability of AT-target protein complexes [77–79]. The heparin-induced conformational change may involve the C-terminal part of the AT molecule and not N-terminal sites alone [80], and Arg229* mutant can cause lack of C-terminal part of the AT molecule just right, which indicates that Arg229* variant may also affect functional domains HBS. The other key sites in the RCL are behind position 229. The reaction site bond of AT is located at the Arg425-Ser426 bond. The presence of Arg425 at P1 position leads to the fairly broad specificity of AT, enabling many coagulation proteinases to be inhibited. Arg229* mutant does not just affect the domain of interaction with FIX of the AT molecule, but also the whole functional domains RCL. Therefore, we hypothesize that this variant may also affect two functional domains, HBS and RCL, with loss of the corresponding functions in the protein.

To further verify the pathogenic mechanism of the two mutations, recombinant Asn460Thrfs*20 and Arg229* recombinant expression vectors were constructed and transfected into 293 T cells. The results show that AT protein expression level was diminished in 293 T cells with the Asn460Thrfs mutant, whereas the AT truncated protein was detected in 293 T cells with the Arg229* mutant; AT protein levels in cell supernatants were also significantly decreased, suggesting that AT protein secretion was impaired. We believe that the discovery of the variant at position 460 causes rupture of the disulfide bond between Cys279 and Cys462. Consequently, the AT protein cannot be properly folded to form a highly conserved spatial structure, and instead forms an unstable structure. As AT has not yet been exported from the cell, it is rapidly degraded intracellularly, resulting in a marked lack of AT in the serum of the proband [81]. In an AT-deficient pedigree study, the presence of the Ile218 mutation was found to result in misfolding and intracellular retention of AT [82], which is similar to the results of our study. The AT truncation protein formed by the Arg229* mutation may affect the anticoagulant activity of AT by losing the functional domains HBS and RCL. The AT truncated protein is retained in the cell owing to impaired secretion and is subsequently rapidly degraded by the proteasome and lysosome, resulting in a decrease in AT content in the plasma. However, bioinformatics analysis software was only used to predict the possible effect of mutation sites on the structure of the AT protein, and experimental evidence is lacking. While cell experiments only verified impaired protein synthesis and secretion, and the specific mechanism is not clear. Further studies are needed to determine which step of the protein synthesis and secretion process is affected by the mutation site.

The choice of genetic test is crucial for improving the detection rate. Using next-generation sequencing and multiple ligand probe amplification of the seven exons and flanking regions of the coding gene, up to 80% of cases of genetic defects can be identified [83]. Whole-genome sequencing may also reveal mutations in the non-coding regions of *SERPINC1*, such as promoters or regulatory sequences. Glycosylation defects can also be detected in *SERPINC1*-negative cells [41].

The challenge in managing patients with AT deficiency is to prevent potentially life-threatening thrombosis, while minimizing the risk of bleeding associated with long-term anticoagulant therapy. Primary prophylaxis is not currently recommended for asymptomatic patients, as long-term anticoagulation increases the risk of fatal bleeding and the risk of fatal VTE is low [84]. Approximately 60% of the thrombotic events in AT-deficient individuals are caused by pregnancy. Patients remain vulnerable to VTE or even fatal thrombotic events, even with adequate anticoagulation during pregnancy and the postpartum period. In high-risk settings, the use of AT replacement therapy reduces this risk [85]. Paradoxically, according to guidelines issued in 2012 by the American College of Chest Physicians, special antithrombotic agents should not be used prophylactically in women with a history of hereditary thrombophilia and pregnancy complications (grade 2C) [86]. Low-molecular-weight heparin (LMWH) has been widely recognized in the literature as a safe and effective alternative to oral anticoagulant therapy (e.g., warfarin) in early and late pregnancy. Unlike unfractionated heparin, LMWH has stronger anti-FXa activity [87]. However, owing to the presence of heparin resistance in many patients with AT deficiency, the therapeutic efficacy of LMWH has not been unanimously recognized, and there are limited data on the use of the ideal dose of LMWH replacement therapy [88].

## Conclusions

In conclusion, two heterozygous mutations of *SERPINC1*, Asn460Thrfs*20 and Arg229*, were identified as the pathogenic mutations, and Asn460Thrfs*20 variant was reported firstly. It was predicted by molecular bioinformatics software that Asn460Thrfs*20 mutation caused the breakage of the intramolecular disulfide bond, further leading to AT misfold, while Alg229* mutation caused the formation of AT truncated protein, which lack of functional domain HBS and RCL. These occur at the level of protein translation. Then it was found that Asn460Thrfs*20 and Arg229* variants could cause disorderly AT synthesis, secretion or intracellular degradation in cells due to protein translation error, and finally led to a decrease in AT levels and activity. However, the specific mechanism need further experimental verification. Our study enriched the mutation database of *SERPINC1* gene, provided some new theoretical basis for gene diagnosis and genetic counseling of patients with AT deficiency, and provided experimental data for subsequent exploration of molecular pathogenesis.

## Supplementary Information

**Additional file 1.** Original Image for Fig. 3c- SERPINC1.

**Additional file 2.** Original Image for Fig. 3c (SERPINC1).

**Additional file 3.** Original Image for Fig. 3c (Tubulin and GAPDH).

**Additional file 4.** Original Image for Fig. 3c-Tubulin and GAPDH.

## Data Availability

The datasets used and/or analyzed during the present study are available from the corresponding author upon reasonable request.

## Abbreviations

AT: Antithrombin
BSA: Bovine serum albumin
LMWH: Low-molecular-weight heparin
RCL: Reactive center loop
VET: Venous thrombosis
