# Supplementary figures and images for: Identification and characterization of two SERPINC1 mutations causing congenital antithrombin deficiency
Source: Thromb J. 2023 Jan 9;21:3. doi: 10.1186/s12959-022-00443-6 (PMC9830717; doi:10.1186/s12959-022-00443-6)

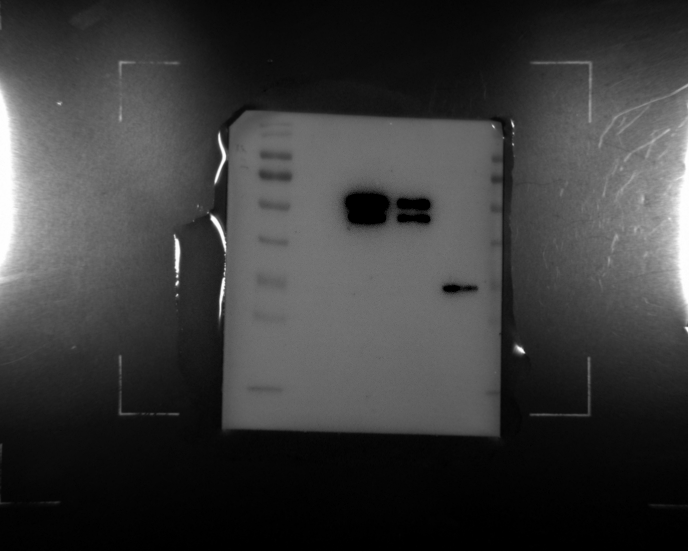

Supplement: Supplementary file 1 — Additional file 1. Original Image for Fig. 3c- SERPINC1. [file 12959_2022_443_MOESM1_ESM.tif]

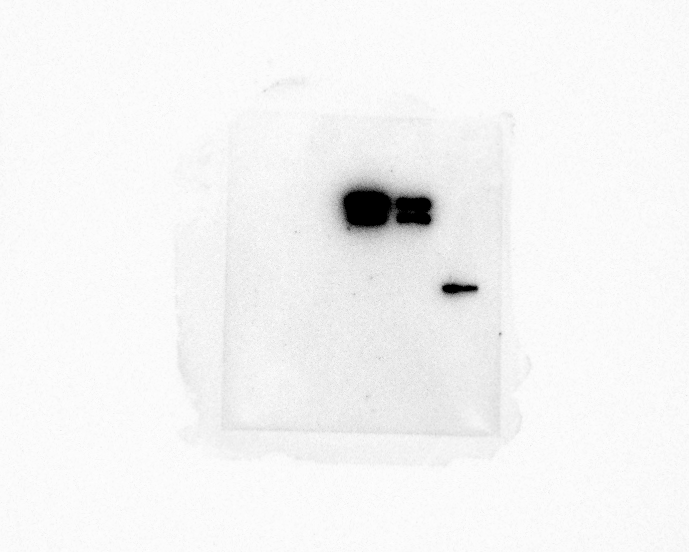

Supplement: Supplementary file 2 — Additional file 2. Original Image for Fig. 3c (SERPINC1). [file 12959_2022_443_MOESM2_ESM.tif]

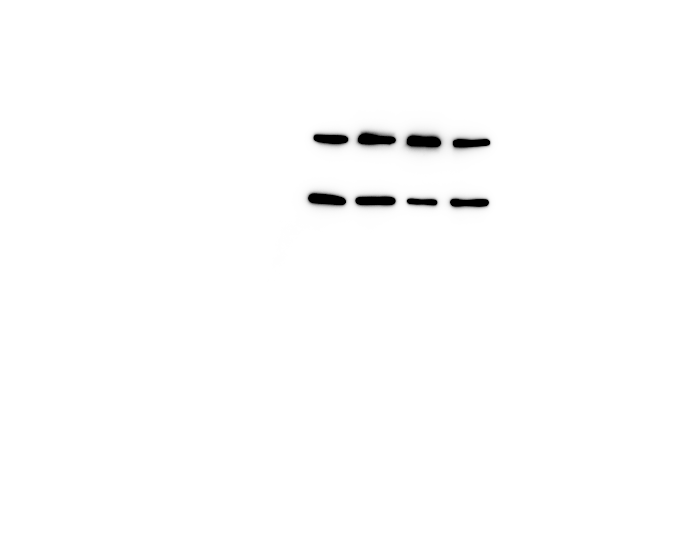

Supplement: Supplementary file 3 — Additional file 3. Original Image for Fig. 3c (Tubulin and GAPDH). [file 12959_2022_443_MOESM3_ESM.tif]

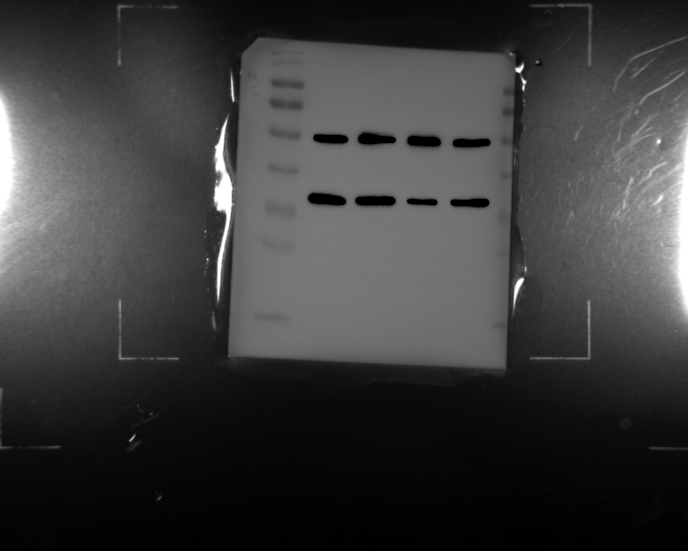

Supplement: Supplementary file 4 — Additional file 4. Original Image for Fig. 3c-Tubulin and GAPDH. [file 12959_2022_443_MOESM4_ESM.tif]
